# Signs of immune dysregulation in second-trimester maternal blood RNA profiles in late-onset preeclampsia

**DOI:** 10.1038/s41598-025-26323-3

**Published:** 2025-11-26

**Authors:** Gamze Yazgeldi Gunaydin, Sini Ezer, Juho Wedenoja, Katri Räikkönen, Juha Kere, Satu Wedenoja, Shintaro Katayama

**Affiliations:** 1 Stem Cells and Metabolism Research Program, University of Helsinki, Helsinki, 00290 Finland; 2 Folkhälsan Research Center, Helsinki, 00290 Finland; 3 Department of Ophthalmology, University of Helsinki and Helsinki University Hospital, Helsinki, 00290 Finland; 4 Department of Psychology, Faculty of Medicine, University of Helsinki, Helsinki, 00014 Finland; 5 Obstetrics and Gynecology, Helsinki University Hospital and University of Helsinki, Helsinki, 00290 Finland; 6 Department of Medicine Huddinge, Karolinska Institutet, Huddinge, 14183 Sweden

**Keywords:** Computational biology and bioinformatics, Medical research, Molecular medicine, Signs and symptoms

## Abstract

Preeclampsia (PE) is a hypertensive disorder that affects 5% of pregnancies. It is characterized by new-onset hypertension and proteinuria after 20 weeks of gestation. We investigated maternal second-trimester peripheral blood gene expression profiles to identify potential biomarkers for predicting late-onset PE. We found significant associations between PE and several inflammatory and immunological pathways, including the JAK-STAT signaling, leukocyte transendothelial migration, systemic lupus erythematosus (SLE), and graft versus host disease (GVHD). Twelve candidate biomarkers were identified, with high predictive power (AUC 0.74–0.87) for late-onset PE. These biomarkers were also associated with various blood cell populations, particularly intermediate monocytes. Our study highlights the role of maternal immune dysregulation in PE and the potential of second-trimester blood RNA profiles in disease prediction.

**Supplementary Information:**

The online version contains supplementary material available at 10.1038/s41598-025-26323-3.

## Introduction

Preeclampsia (PE) is a hypertensive pregnancy complication, characterized by new-onset hypertension and proteinuria or other related symptoms after 20 weeks of gestation^1^. PE affects approximately 5% of all pregnancies, with higher prevalences in developing countries^1,2^. This complex disorder mostly affects first-time mothers and is influenced by several maternal risk factors including obesity, cardiovascular disease, or systemic lupus erythematosus (SLE)^3^. Approximately 70% of PE develops after 34 weeks of gestation, defined as late-onset PE^4^.

Although only the delivery of the placenta cures PE, its underlying placental etiology remains incompletely understood. PE involves pre-clinical and clinical stages^5^. First, poor placentation and/or placental malperfusion causes syncytiotrophoblast stress, leading to the release of pro-inflammatory and anti-angiogenic agents into the maternal circulation^3,6^. Second, placental stress promotes generalized vascular inflammation and new-onset hypertension and proteinuria or other signs of end-organ dysfunction in the mother^5^. While early-onset PE is linked to poor placentation and fetal growth restriction, late-onset PE is more related to maternal risk factors.

Prediction of PE before the onset of clinical syndrome could improve maternal and fetal outcomes by enabling timely interventions, reducing complications, and lowering healthcare costs^7^. Current risk assessment involves maternal history and combinations of biomarkers such as mean arterial blood pressure, uterine artery pulsatility index, and placental growth factor (PlGF)^8,9^ These are, however, only useful in predicting early-onset PE. To discover potential biomarkers for presymptomatic prediction of late-onset PE, we studied gene expression profiles of maternal peripheral blood samples taken at 20–24 weeks of gestation. We found that several inflammatory and immunological pathways were associated with late-onset PE, weeks before the onset of clinical syndrome. These included the JAK-STAT signaling, leukocyte transendothelial migration, SLE, and graft versus host disease (GVHD) pathways. Moreover, we identified 12 candidate second-trimester biomarkers to predict late-onset PE.

## Materials and methods

### Sample collection and clinical characteristics

Samples were collected as part of the InTraUterine sampling in early pregnancy (ITU) cohort study at the Helsinki University Hospital, Finland. Data on cohort collection and sampling have been described^10^. The study protocol was approved by the Coordinating Ethics Committee of the Helsinki and Uusimaa Hospital District (269/13/03/00/09). Register data had been merged with approval from the register authority (THL/887/14.02.00/2021). All participants gave their signed informed consent during the recruitment. All methods were carried out in accordance with relevant guidelines and regulations.

Peripheral blood samples were collected between gestational weeks 20 and 24. A total of 64 samples, including 12 PE and 52 normotensive pregnancies (NP) were selected. All women with PE had late-onset disease ≥ 34 weeks, diagnosed using the most recent criteria^11^. The following maternal demographic and clinical data were recorded: maternal age at delivery, parity, hypertension, diabetes, pre-pregnancy body mass index (BMI), obesity status (BMI ≥ 30 kg/m^2^ denoting obesity), gestational age at birth, PE status (PE or NP), sex of the baby, birth weight, and placental weight. Although diabetes and obesity status were recorded, they were not included in the analyses because none of the normotensive women were obese or had diabetes. Clinical characteristics were tabled using Table 1 package (version 1.4.3)^12^ in R (version 4.2.2)^13^. Group comparisons were performed using t-test for parametric (*p* > 0.05, Shapiro-Wilk test), Mann-Whitney *U* test for non-parametric (*p* ≤ 0.05), and Chi-square test for categorical variables.

**Table 1:** Clinical characteristics of the study population. Shown are the clinical characteristics of 49 pregnant women.

	Preeclampsia (PE) (*n* = 10)	Normotensive pregnancy (NP) (*n* = 39)	*p*-value
Maternal age at delivery (years)			
Mean (SD)	35 (± 3.0)	34 (± 5.0)	0.54
Maternal pre-pregnancy body mass index (BMI)			
Mean (SD)	23 (± 3.0)	22 (± 1.6)	0.12
Maternal pre-pregnancy body mass index (BMI) categories			
Normal weight	7 (70%)	39 (100%)	0.01
Obese	3 (30%)	0 (0%)	
Number of previous pregnancies leading to delivery (Parity)			
0	6 (60%)	19 (49%)	0.82
1	3 (30%)	15 (38%)	
≥2	1 (10%)	5 (13%)	
Gestational age at birth (weeks)			
Mean (SD)	38 (± 1.5)	40 (± 1.3)	0.01
Child sex			
Boy	5 (50%)	18 (46%)	1
Girl	5 (50%)	21 (54%)	
Child birth weight (grams)			
Mean (SD)	3100 (± 470)	3500 (± 360)	0.02
Library			
Library 1	2 (20%)	18 (46%)	0.25
Library 2	8 (80%)	21 (54%)	
RNA integrity number (RIN)			
Mean (SD)	8.7 (± 0.39)	8.3 (± 0.49)	0.02

## STRT library preparation and the sequencing

RNA was extracted from maternal peripheral blood samples, preserved in PAXgene Blood RNA Tubes (QIAGEN), using PAXgene Blood RNA kit (QIAGEN). A modified 5’-end RNA-seq method, STRT, with 8-bp unique molecular identifiers (UMIs)^14^ was applied by combining GlobinLock technique^15^. For the sample identification, 6-bp barcode sequences were included in the primers. Two STRT libraries, each having 48 samples and water as non-template controls (NTC), were sequenced with Illumina NextSeq 500 System, High Output (75 cycles, single-end). The sample layout was balanced for PE and NP status between the two libraries. In total, 97 spike-ins from the ERCC RNA Spike-in mix were tested.

## Preprocessing and the quality control of the STRT libraries

STRTN pipeline (https://github.com/gyazgeldi/STRTN, commit e16a6d1)^16^ was employed for this step. The raw data were aligned to the hg38 reference genome with GENCODE v43 basic annotation (wgEncodeGencodeBasicV43), which was downloaded from the UCSC Genome Browser (https://genome.ucsc.edu/)^17^; then a count matrix for protein-coding genes, and several quality control metrics were obtained. Outlier samples were assessed based on quality metrics, including mapped reads, spike-in reads, spike-in 5’-end rate, mapped rate, mapped/spike-in ratio, and coding 5’-end rate, and identified as values outside the interquartile range-based threshold range (values below than Q1–1.5×IQR or above than Q3 + 1.5×IQR) in boxplots.

## Reduction of technical bias and noise in the gene expression profiles

Genes with counts below five were filtered out, and library specific biases between the two STRT libraries were corrected using library_bias_correction function from NBGLM-LBC (Negative Binomial Generalized Linear Model – Library Bias Correction) package^18^ in R (version 4.2.2). Depth files were generated from BAM files using samtools^19^, followed by processing and classification by library. Thereafter, feature selection test^20^ was applied to identify fluctuating (highly variable or informative) genes to reduce technical noise. This test calculated the squared coefficient of variation for each gene and spike-ins in the library bias corrected and spike-in normalized count matrix. Technical variation was evaluated by analyzing the fluctuations in spike-in RNA levels across the cells, allowing for the comparison of each gene’s expression changes with predicted variations from the spike-in controls. Genes with adjusted p-values < 0.05 were considered fluctuating, indicating a higher gene-to-spike-in ratio in the squared coefficient of variation. These genes were kept, while those with adjusted p-values > 0.05 were filtered out. All spike-ins were kept without considering fluctuation as minor fluctuations reflected technical noise rather than biology.

## Dimensionality reduction and visualization of gene expression profiles

Principal Component Analysis (PCA) was applied for dimensionality reduction and clustering and Uniform Manifold Approximation and Projection (UMAP) was used for two-dimensional visualization of the library bias corrected, fluctuating genes expression data using the Seurat package (version 5.0.1)^21^ in R, followed by spike-in normalization with offset and log transformation. PCAtools package (version 2.10.0)^22^ in R was then used, by removing the lower 20% of variables that explained minimal variance, to extract the most informative features and visualize the Pearson’s correlations between principal components (PCs) and clinical characteristics using eigencorplot and pairplots.

### Identification of differentially expressed genes

Differential gene expression analysis (DGE) analysis was performed using the DESeq2 package (version 1.38.3)^23^. The analysis was based on the library bias corrected count matrix. Genes with fewer than 5 counts across the smallest group size (*n* = 10) were filtered out prior to analysis. Spike-in normalization was applied using all 88 non-zero expressed spike-ins as control genes in the DESeq2 function estimateSizeFactors(), where a logical vector was provided to specify the spike-ins. The design formula was ~ Condition (PE vs. NP). The Wald test was used, and p-values were adjusted for multiple testing using Benjamini-Hochberg method (α = 0.05). Log2 fold-change (log2FC) values were then used to show how the genes in the enriched pathways change between PE and NP, using the Pathview package (version 1.38.0)^24^.

## Pathway and cell type enrichment analysis

Gene set enrichment analysis (GSEA)^25^ was performed using the curated pathway collection CP: KEGG_LEGACY^26–29^ to identify significant pathways associated with PE. Using the fgsea package (version 1.24.0)^30^ in R, genes were ranked based on PC loadings, reflecting their contributions to each principal component (PC). Pathways enriched at the top or bottom of the ranked gene list were identified using normalized enrichment scores (NES) degree, and expression trend. NES values were then calculated to determine the association between gene sets and PCs. Leading genes, which are the most influential in the biological processes and contribute to the observed enrichment signal, were also detected to highlight key genes associated with PE or distinguishing PE samples. For each enriched pathway, spike-in normalized gene expression values of member genes were transformed into Z-score, scaled, and visualized as heatmaps with ComplexHeatmap package (version 2.14.0)^31^. Additionally, GSEA was performed using only the fluctuating genes to identify enriched cell types in the PE samples compared to NP samples. For this, a custom gene set was prepared using the human blood cells GSE149938 dataset^32^. This dataset originally contained 32 cell types. However, we focused on circulating blood cells and excluded hematopoietic stem and progenitor cells, including hematopoietic stem cells, granulocyte-monocyte progenitors, B cell and natural killer cell progenitors, multi-lymphoid progenitors, lymphoid-primed multipotent progenitors, megakaryocyte-erythroid progenitors, common myeloid progenitors, and multipotent progenitors. To identify cell-type-specific markers, differentially expressed genes (log2FC > 2.32, padj < 0.05) were detected using Seurat (version 5.0.1). Marker genes were then compiled into a GMT file for GSEA, enabling enrichment analysis of the immune cell types associated with PE.

## Prediction of PE status using logistic regression analysis

Spike-in normalized fluctuated data and sample metadata were combined, resulting in 6,982 variables. Logistic regression models were then fitted to predict PE status for each variable. Key metrics, including Area Under the Curve (AUC), sensitivity, specificity, and accuracy along with their confidence intervals at the fixed 10% false-positive rate, were calculated using pROC (version 1.18.5)^33^ and ROCR (version 1.0.11)^34^ packages in R. Confidence intervals (CI) were calculated at the 95% confidence level. Statistical significance of each ROC curve was evaluated by testing whether the AUC was greater than 0.5, with p-values calculated using the Mann–Whitney U test, which is equivalent to the DeLong test. Results were filtered to highlight variables with sensitivity above 0.65, focusing on those with the highest diagnostic potential. The gene expression levels of these highlighted variables were visualized in PE and NP using violin plots with the VlnPlot function from the Seurat package. Differences in gene expression between these two conditions were assessed using the two-sided Wilcoxon test.

## Results

**Fig. 1:**
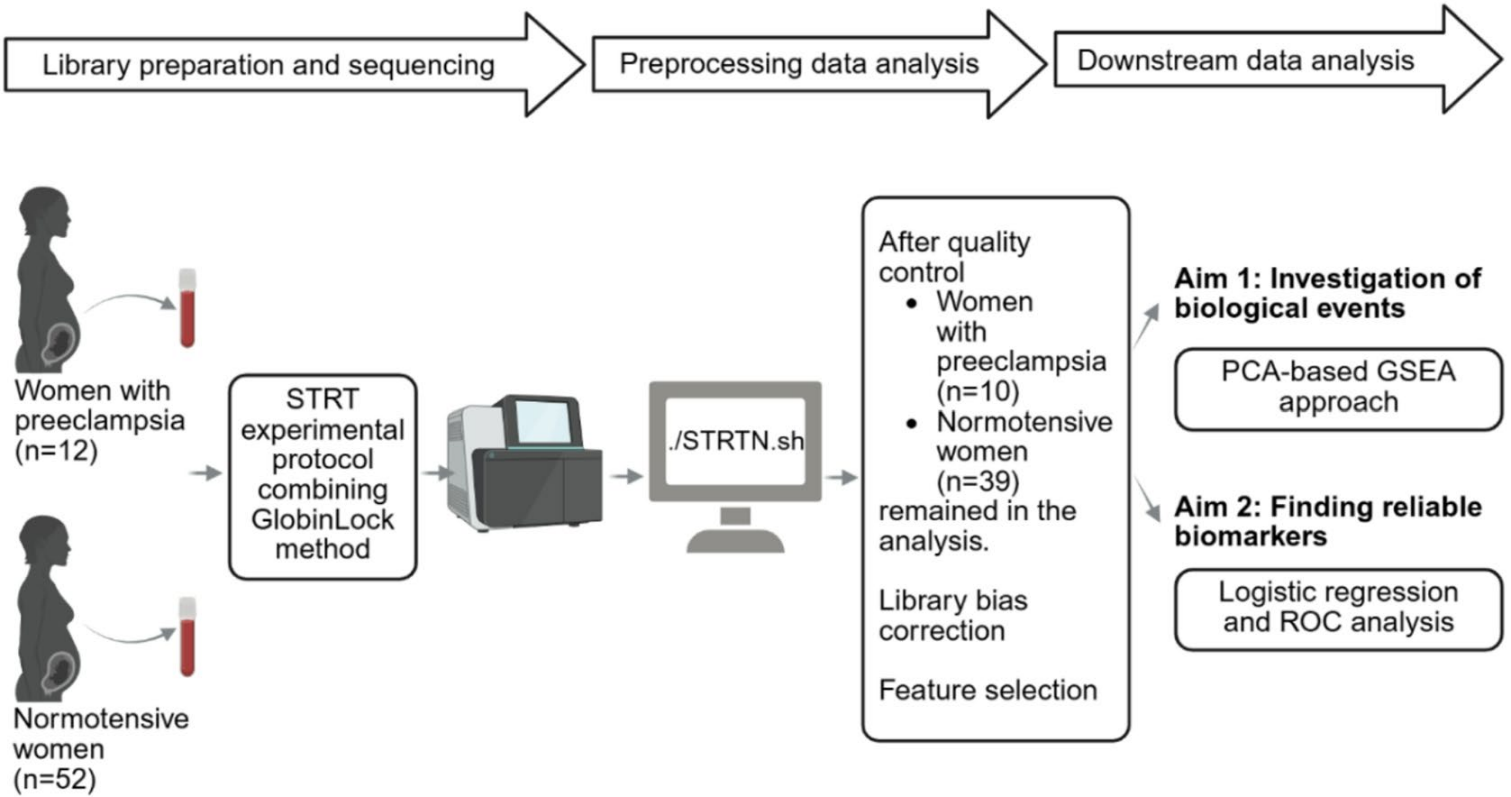
Flow chart of the study.

An overview of the study design is shown in Fig. 1, and demographics of the study population in Table 1.

For this study, we specifically selected late PE cases and uneventful pregnancies, for which we had biological samples available and taken at the same gestational age. While the overall prevalence of gestational hypertension was 7% in the whole ITU cohort^10^, our cohort included 12 (19%) PE cases among the total of 64 women. Mean maternal ages at delivery, parity, and pre-pregnancy BMIs in early pregnancy were similar between the groups. However, 3 (30%) of PE women were obese while all controls had normal weight. Moreover, PE women delivered at earlier gestational age than the NP group, and as expected, their neonates had lower mean birth weights^35^. One patient with PE delivered at 35 weeks, while all other deliveries occurred at or after 37 weeks. After the quality control (Supplementary Fig. 1), two PE and 13 NP samples were excluded as outliers, leaving 49 (10 PE and 39 NP) samples from two unbiased libraries (Supplementary Fig. 1) for downstream analyses.

### Immune activity and inflammation pathways associated with late-onset PE at the presymptomatic phase

To identify transcriptomic alterations and pathway-level changes associated with late-onset PE at the presymptomatic phase, we started with the initial dataset detecting signals for 19,670 genes, including 97 spike-ins. After filtering steps, 12,670 genes and 91 spike-ins remained in the analysis. After the library bias correction and feature selection, we obtained the final dataset of 6,804 genes, including the spike-ins, for downstream analyses.

**Fig. 2:**
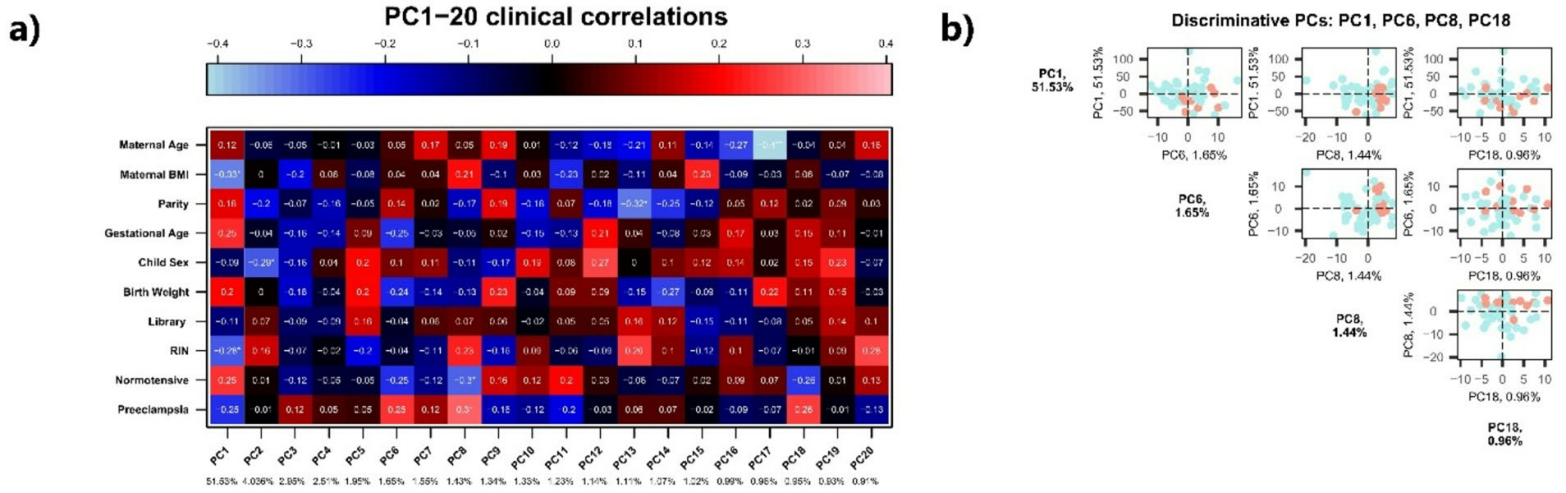
Transcriptomic alterations in second-trimester maternal blood samples from women with late-onset preeclampsia during the presymptomatic phase. (a) PCA-correlation plot demonstrates that PC1, PC6, PC8, and PC18 are correlated (Pearson’s *r* ≥ 0.25) with preeclampsia (PE). Percentages of variation that each component represents are shown at the bottom line. (b) Biplot shows that PC1, PC6, PC8, and PC18 only partially distinguish PE and normotensive (NP) samples. PE samples are shown in red and NP in blue.

Conventional DEG analysis did not reveal differentially expressed genes between PE and NP women (adjusted p-values were ~ 0.9). Using standard DESeq2 settings with Benjamini-Hochberg correction (α = 0.05), 771 genes showed nominal significance (*p* < 0.05), but none remained significant after multiple testing adjustment. Since no clear signals were detected at the single-gene level, we therefore applied a PCA-GSEA approach to improve sensitivity^36^. This approach reduces the effect of individual variation by grouping correlated genes into principal components and then testing for enrichment at the pathway level, rather than focusing on single-genes differences as done in DEG. Using this approach, we identified significant associations between clinical characteristics and four PCs that strongly correlated with PE (Fig. 2a). Of them, PC1 explained 51.53% of the total variation and was negatively correlated with PE (Pearson’s correlation coefficient: −0.25). PC6, PC8, and PC18 explained 0.95 to 1.95% of the variation and were positively correlated with PE (Pearson’s correlation coefficients ≥ 0.25) (Fig. 2a). PE and NP samples were only partially separated by the PCs: PE samples were in the negative area of PC1 and positive area of other PCs (Fig. 2b).

**Fig. 3:**
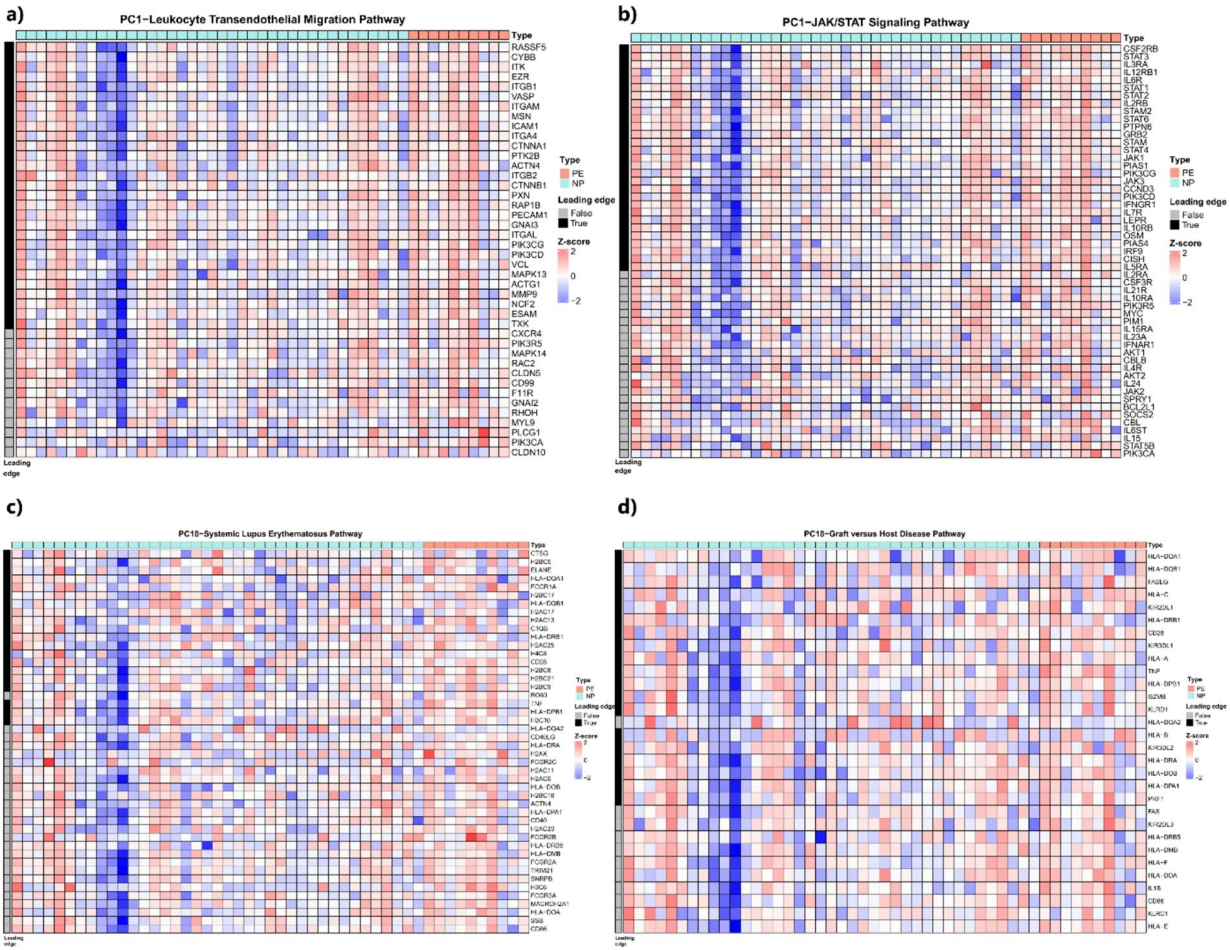
Heatmaps showing differential second-trimester pathway activation in women with late-onset preeclampsia and normotensive women. Shown are the major upregulated pathways in preeclampsia (PE) related to (**a**) leukocyte transendothelial migration, (**b**) JAK-STAT signaling, (**c**) systemic lupus erythematosus (SLE) and (**d**) graft versus host disease pathways. Columns represent samples (ordered by rotated values in each principal component, PC), and rows represent pathway genes (ordered by loading values in each PC), with the left indicating leading genes. Red color indicates upregulation, and blue indicates downregulation, normalized across preeclampsia (red) and normotensive pregnancies (blue).

Using the KEGG Legacy dataset, we found that the genes highly contributing to the PE-correlated PC1, PC6, and PC18 were significantly enriched in several pathways (Table 2), whereas no pathways were enriched for PC8. For PC1, the leukocyte transendothelial migration pathway, JAK-STAT signaling pathway, and Vibrio cholerae infection pathway were significantly negatively enriched (Table 2). As these pathways contributed negatively to PC1, and PC1 was negatively correlated with PE (Fig. 2a), these pathway genes were upregulated in PE (Fig. 3a-b, Supplementary Figs. 2–4). As for PC6, being positively correlated with PE, the antigen processing and presentation pathway, and asthma pathway were negatively enriched (Table 2), indicating that their member genes were downregulated in PE (Supplementary Figs. 5 and 6). As for PC18, being also positively correlated with PE, the SLE pathway, GVHD and antigen processing and presentation pathways were positively enriched (Table 2), indicating that their member genes were upregulated in PE (Fig. 3c-d, Supplementary Figs. 7–9). Interestingly, the antigen processing and presentation pathway was negatively enriched in PC6 and positively enriched in PC18, reflecting variations captured by each PC and the influence of other pathway-specific genes, detailed in Fig. 3 and Supplementary Figs. 5 and 7.

**Table 2:** Pathways associated with transcriptomic alterations in second-trimester maternal blood samples before late-onset preeclampsia. Shown are enriched pathways with KEGG pathway ID, padj < 0.05, and their corresponding normalized enrichment scores.

PCs​	Enriched pathway​	Pathway ID​	padj	NES	Direction of change in women with preeclampsia (PE)
PC1​	Leukocyte trans-endothelial migration​	hsa04670	3.12 × 10^− 6^	−2.83	Upregulated
	JAK-STAT signaling pathway​	hsa04630	4.56 × 10^− 3^	−2.26	Upregulated
	Vibrio cholerae infection​	hsa05110	2.71 × 10^− 2^	−2.10	Upregulated
PC6​	Antigen processing and presentation​	hsa04612	1.49 × 10^− 2^	−1.87	Downregulated
	Asthma​	hsa05310	1.49 × 10^− 2^	−2.08	Downregulated
PC18​	Systemic lupus erythematosus​	hsa05322	4.52 × 10^− 3^	2.06	Upregulated
	Graft versus host disease​	hsa05332	8.25 × 10^− 3^	2.03	Upregulated
	Antigen processing and presentation​	hsa04612	2.74 × 10^− 2^​	1.85	Upregulated

PC = Principal Component, Pathway ID = KEGG Pathway Identifier, padj = Adjusted p-value, NES = Normalized Enrichment Score.

### High-AUC biomarkers for late-onset PE prediction suggesting intermediate monocytes enrichment in maternal second-trimester blood RNA profiles

**Fig. 4:**
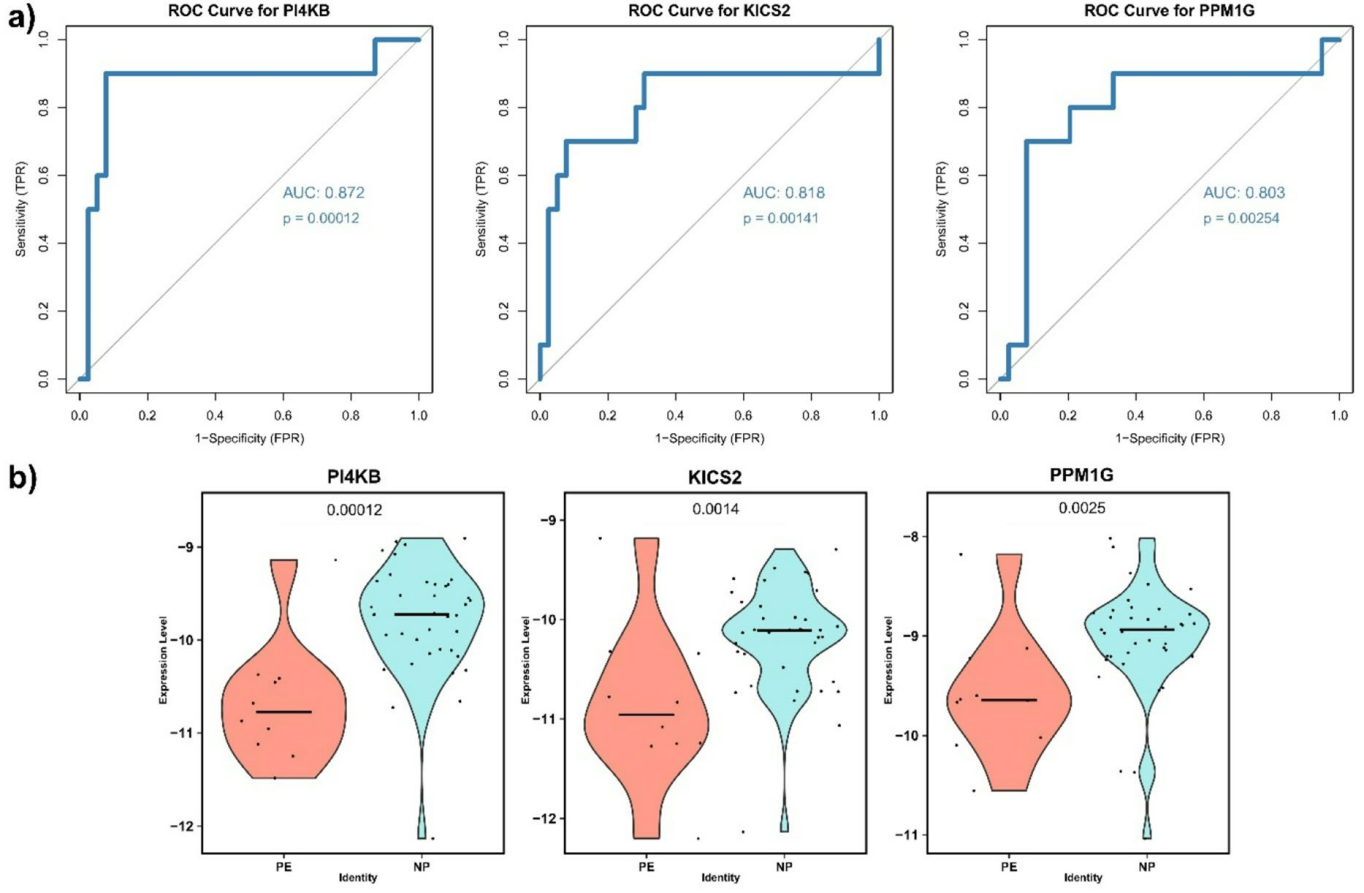
Performances of three candidate genes for late-onset preeclampsia prediction. (a) ROC curves show the performance of selected biomarkers in distinguishing preeclampsia (PE) and normotensive pregnancies (NP) with false positive rate (FPR) on the x-axis and true positive rate (TPR) on the y-axis. The area under curve (AUC) values and corresponding p-values (testing whether AUC > 0.5) provide a measure of the diagnostic power of each biomarker. (b) Violin plots showing variation, with p-value from the two-sided Wilcoxon test shown above, in gene expression of the same biomarkers between PE and NP. Horizontal lines indicate median expression.

After observing pathway alterations associated with late-onset PE in the second-trimester maternal blood samples, we searched for potential marker genes for PE prediction. We performed logistic regression analysis followed by ROC analysis. We found that several genes distinguished PE and NP with AUC values of 0.74 to 0.87 (Table 3), as visualized in Fig. 4a and Supplementary Fig. 10. Their expression levels in PE and NP samples are shown in Fig. 4b and Supplementary Fig. 10. As shown in these figures, the identified biomarkers exhibited higher expression in NP compared with PE samples. Despite the high AUC and sensitivity values, the small sample size resulted in wide confidence intervals for sensitivity estimates (Table 3). Moreover, the genes identified through the ROC analysis, with a sensitivity threshold set at 0.65, were not directly involved in the pathways detected by the PCA analysis (Table 3).

**Table 3:** Performances of 12 candidate genes in disease prediction in maternal second-trimester blood samples of women with late-onset preeclampsia and normotensive women. Shown are area under curve (AUC) values, sensitivity, accuracy, and their 95% confidence intervals (CI) at the fixed 10% false-positive rate for the top biomarkers.

Variable​	AUC​	AUC CI​	Sensitivity​	SensitivityCI​	Accuracy​
PI4KB (Phosphatidylinositol 4-Kinase Beta)	0.87​	0.701–1​	0.9​	0.2–1​	0.9​
KICS2​ (KICSTOR Subunit 2)	0.81​	0.621–1​	0.7​	0.2–0.9​	0.85​
PPM1G​ (Protein phosphatase 1G)	0.80​	0.615–0.99​	0.7​	0–0.9​	0.85​
HIPK3​ (Homeodomain Interacting Protein Kinase 3)	0.79​	0.606–0.989​	0.7​	0.2–0.9​	0.85​
TRIM68​ (Tripartite Motif Containing 68)	0.79​	0.59–0.995​	0.7​	0.2–0.9​	0.85​
SFI1​ (SFI1 centrin binding protein)	0.78​	0.576–0.998​	0.7​	0.1–0.9​	0.85​
APOL6 (Apolipoprotein L6)​	0.78​	0.576–0.988​	0.7​	0–1	0.85​
ARSG​ (Arylsulfatase G)	0.77​	0.552–0.997​	0.7​	0.1–0.9​	0.85​
PITRM1​ (Pitrilysin metallopeptidase 1)	0.77​	0.551–0.992​	0.7​	0.1–0.9	0.85​
USO1​ (USO1 vesicle transport factor)	0.76​	0.552–0.981​	0.7​	0.1–0.9​	0.85​
RAB11B​ (Member RAS oncogene family)	0.76​	0.54–0.983​	0.7​	0.2–1	0.85​
FAM117A​ (Family with sequence similarity 117 member A)	0.74​	0.517–0.97​	0.7​	0–0.9​	0.85​

AUC = Area Under the Curve, CI = Confidence Interval.

**Table 4:** Genes associated with different cell populations for late-onset PE prediction. Shown are enriched cell type signatures with padj < 0.05 and their corresponding normalized enrichment scores.

Ranking method	Cell type	padj	NES	Predictive role in women with preeclampsia (PE)
AUC	Erythrocytes	4.02 × 10^− 12^	−4.53	Negative
	Progenitor neutrophils	1.59 × 10^− 6^	−3.28	Negative
	Myelocytes	1.72 × 10^− 6^	−3.21	Negative
	Intermediate monocytes	4.08 × 10^− 3^	2.21	Positive
	Metamyelocytes	1.09 × 10^− 2^	−1.96	Negative
	Mature neutrophils	2.80 × 10^− 2^	−1.77	Negative
	Regulatory B cells	3.81 × 10^− 2^	−1.79	Negative

padj = Adjusted p-value, NES = Normalized Enrichment Score.

Since the candidate markers (Table 3) were not part of known pathways, we further tested whether they were associated with different blood cell populations. Specifically, we analyzed a total of 24 circulating blood cell types, ranking biomarkers separately based on AUC to assess their ability to distinguish PE from NP, and on sensitivity to assess their ability to detect PE. We found that the sensitivity-based ranking did not identify any significantly enriched cell types associated with PE samples. In contrast, the AUC-based ranking identified significant enrichment in human blood cell marker genes for erythrocytes, progenitor neutrophils, myelocytes, intermediate monocytes, metamyelocytes, mature neutrophils, and regulatory B cells (Table 4). Among them, intermediate monocytes had a positive NES, indicating that these cell-type marker genes were most effective in distinguishing PE samples. Conversely, other cell types had negative NES values, suggesting a lower ability to distinguish PE from NP.

## Discussion

Tools for identifying late-onset PE at the presymptomatic phase, before the onset of maternal syndrome, are currently lacking. To address this challenge, we utilized maternal second-trimester blood samples to explore early alterations in RNA expression profiles before late-onset PE. We found that subtle changes in RNA expression profiles arise weeks before the onset of the clinical syndrome. Notably, PE-associated changes were related to leukocytic immune surveillance and maternal immunity homeostasis. Based on these observations, we found 12 potential candidate genes for PE prediction.

In our study, second-trimester maternal RNA expression profiles revealed alterations in several pathways among women with subsequent development of late-onset PE. These included upregulation of genes of the leukocyte transendothelial migration pathway that regulates immune surveillance through the movement of immune cells across the endothelium^37^. This pathway has previously been linked to immune activation during pregnancy, and additionally, to infection-related and immunological PE subtypes in placental transcriptome^36^. Upregulation of leukocyte transendothelial migration pathway is in agreement with prior findings that indicate maternal endothelial and leukocyte activation in PE^38^. To support the biological role of this pathway in PE, leukocyte migration genes are even candidate predictors of gestational age in the peripheral blood of normal pregnancies^39^. Similarly, genes in the JAK/STAT pathway, a critical mediator of cytokine and growth factor signaling, were upregulated in late-onset PE, further supporting alterations in immune responses or homeostasiss^40^. Thus, there are two possible interpretations: one involves the activation of maternal inflammatory responses already at the presymptomatic phase of PE, and the other suggests primary maternal immune dysregulation in pregnancies predisposed to subsequent development of PE. To support the role of primary immune aberration, genes in the SLE pathway were upregulated among women with subsequent PE, even if none of the participants had SLE. Among other connective tissue diseases, SLE is a chronic inflammatory disease with altered immune homeostasis^41^, and a considerable risk factor of PE^42^. Interestingly, one of the leading genes of the SLE pathway, cathepsin G (CTSG), shows upregulation in normal pregnancies versus non-pregnant controls^43^ of first-trimester maternal plasma, and contributes to the processing of antigens and autoantigens^44^, proposing its primary role during human pregnancy. As a potential sign of immune rejection, genes in the GVHD pathway were among those upregulated in PE, supporting the role of enhanced maternal immune responses in the PE pathogenesis^45^.

The candidate biomarkers that we identified have not been previously reported in association with PE. Among the top genes, some are directly linked to PE or placental processes. PI4KB (Phosphatidylinositol 4-Kinase Beta) is a lipid metabolism enzyme involved in vascular morphogenesis through the control of fibronectin secretion in endothelial cells^46^. As vascular remodeling is essential for placentation, and maternal endothelial dysfunction is a hallmark of PE^47^, its upregulation in late-onset PE may reflect early signs of endothelial dysfunction. KICS2 (KICSTOR subunit 2) regulates autophagy via mTORC1 signaling^48^. Autophagy is an important process for placentation, especially for trophoblast invasion^49^, and impaired placental autophagy has been observed in PE^50^. Autophagy also contributes to monocyte-macrophage differentiation^51^, suggesting that the PE-related changes in monocytes populations observed previously^52–54^ and in our study might be at least partially related to these alterations. HIPK3 (particularly its circular isoform circHIPK3) is also to be noted, as it is downregulated in preeclamptic placentas, with functional studies showing reduced trophoblast proliferation, migration and invasion^55,56^.

Among the other identified genes, many link to immune or blood cell functions. PPM1G is a phosphatase that regulates multiple cellular processes, including mRNA splicing, DNA damage response, and cell cycle progression. Its elevated expression has been associated with poor prognosis in hepatocellular carcinoma and shown to correlate with immune cell infiltration and NF-κB–mediated inflammatory signaling^57^. RAB11B, a Ras-like small GTPase, is known to regulate intracellular trafficking processes and immune receptor signaling^58^. It has also been linked to autophagy regulation and macrophage polarization, thereby influencing inflammatory responses^59^. FAM117A, also known as C/EBP induced protein, contributes to B-cell to myeloid differentiation, macrophages, and granulocytes in blood cell development^60^ and NK cell metabolism in cancer^61^. APOL6 has been associated with ferroptosis-related pathways and increased infiltration of immune cells such as CD8 + T cells, macrophages, and dendritic cells in cancer^62^, and exosomal regulation of APOL6 was shown to affect endometrial stromal cell survival^63^. TRIM68 is an E3 ubiquitin ligase involved in immune regulation and apoptosis in cancer^64,65^. In osteosarcoma, its expression correlated with increased memory CD4 T cells and Tregs, and decreased mast cells^66^, suggesting a role in adaptive immune responses. Although direct evidence from blood cells or immune cells is limited, these findings support a potential role for immune regulation.

Other identified genes have been studied mainly in placental or general cellular processes. SFI1 is a centriolar protein essential for centriole stability and ciliogenesis^67^. ARSG encodes a lysosomal sulfatase linked to metabolic stress and proinsulin levels, a metabolic trait associated with obesity^68^ and a known risk factor for PE. PITRM1, a mitochondrial metallopeptidase, is expressed in human placenta^69^ and has been reported to be abnormally upregulated in mouse nuclear transfer placentas^70^. USO1 is a vesicle transport factor that mediates ER-to-Golgi trafficking and is part of the phosphatidylinositol 3-kinase (PtdIns3K) complex, essential for autophagy and autophagosome assembly in intestinal epithelial cells^71^. While these genes are involved in placental or cellular pathways, their specific relevance in maternal blood and late-onset PE remains to be clarified.

While we investigated the cellular mRNAs in peripheral blood, many previous studies used cell-free molecules, not only RNAs but also DNAs^7^. Those approaches measure cell content primarily leaked from damaged cells. For example, Farina et al.^72^ could predict (both early- and late-onset) PE using cell-free mRNAs of Fms Related Receptor Tyrosine Kinase 1 (FLT1) at 10–14 weeks of gestation; the detection rate at a 5% false positive rate was 72.3%. Because *FLT1* gene is highly expressed in placental trophoblasts and endothelial cells throughout the body^73^, and secreted in soluble forms, its high circulating levels make it an ideal marker for disease detection when the tissue breakdown occurs. In contrast, our approach measures cellular mRNAs originating primarily from intact cells of the peripheral blood. Therefore, the placental status cannot be assessed by our method but instead, our study provides data on maternal immune status and dysregulation based on leukocyte-derived mRNA alterations.

The main limitation of our study was the moderate number of samples. A larger series would have been ideal due to the heterogeneous nature of late-onset PE^6^, and the complexity of PE pathogenesis^1^. Furthermore, we had neither data on PE subtypes nor the severity of PE. These limitations may also explain why conventional DEG analysis failed to identify significant single-gene changes, partly due to the subtle variation observed at the presymptomatic phase of late-onset PE. Therefore, pathway-level approaches such as PCA-GSEA are more suitable in such cases. Genes that interact with each other for a function tend to co-regulate, and PCA groups co-regulated genes into principal components, so that genes in the same pathway could be enriched by PCA. Unlike DEG, Broad GSEA evaluates all genes, including those without significant changes at the single-gene level. Therefore, if the pathway-level alteration is consistent across patients with the same disease, the combination of PCA and GSEA can detect it. The present study detected several pathways as early signals of late-onset PE using this approach, and the robustness must be validated in other cohorts. However, our study utilized several variables that affected the expression profiles, including maternal pre-pregnancy BMI (PC1 of Fig. 2), fetal gender (PC2), or gestational diabetes (PC3), suggesting that future studies should not only include larger sample sizes but also account for these characteristics. Unfortunately, we had no longitudinal samples or samples at the diagnosis, limiting the conclusions of this study. As a strength of the study, however, the wide coverage of protein-coding genes allowed us to search for potential biomarkers for disease prediction.

Collectively, these results provide novel insights into the complex pathogenesis of PE and its prediction at the presymptomatic phase. Our study suggests that maternal immune dysregulation is an early sign of the disease and is measurable by maternal blood RNA expression profiles. Future studies are needed to characterize and stratify these findings in larger series and clinical settings.

## Supplementary Information

Below is the link to the electronic supplementary material.

Supplementary Material 1

## Data Availability

The datasets generated and/or analyzed during the current study are not publicly available due to restrictions imposed by the Finnish Data Protection Act and EU GDPR legislation. Data are stored at HUS Academic closed access computing environment. An interested researcher can obtain a de-identified dataset after having obtained an approval from the ITU study board and data controller. Data requests may be subject to further review by the national register authority and by the ethical committees. Any requests for data use should be addressed to the corresponding author.
