# Supplementary material for: Signs of immune dysregulation in second-trimester maternal blood RNA profiles in late-onset preeclampsia
Source: Sci Rep. 2025 Nov 26;15:42233. doi: 10.1038/s41598-025-26323-3 (PMC12658047; doi:10.1038/s41598-025-26323-3)
Supplement: Supplementary file 1 — Supplementary Material 1 [file 41598_2025_26323_MOESM1_ESM.pdf]

Supplementary Information

Signs of immune dysregulation in second-trimester maternal blood RNA profiles in late-onset preeclampsia

Gamze Yazgeldi Gunaydin (ORCID: 0000-0002-2041-7300)[1,2,6*], Sini Ezer (ORCID: 0000-0002-1570-6167)[1,2], Juho Wedenoja (ORCID: 0000-0002-6155-0378)[3], Katri Räikkönen (ORCID: 0000-0003-3124-3470)[4], Juha Kere (ORCID: 0000-0003-1974-0271)[1,2,6], Satu Wedenoja (ORCID: 0000-0003-0212-4851)[1,2,5], Shintaro Katayama (ORCID: 0000-0001-7581-5157)[1,2,6*]

[1] Stem Cells and Metabolism Research Program, University of Helsinki, 00290 Helsinki, Finland
[2] Folkhälsan Research Center, 00290 Helsinki, Finland
[3] Department of Ophthalmology, University of Helsinki and Helsinki University Hospital, 00290, Helsinki, Finland
[4] Department of Psychology, Faculty of Medicine, University of Helsinki, 00014 Helsinki, Finland
[5] Obstetrics and Gynecology, Helsinki University Hospital and University of Helsinki, 00290, Helsinki, Finland
[6] Department of Medicine Huddinge, Karolinska Institutet, 14183 Huddinge, Sweden

* Correspondence: gamze.yezgeldi@helsinki.fi, shintaro.katayama@folkhalsan.fi

Supplementary Figures

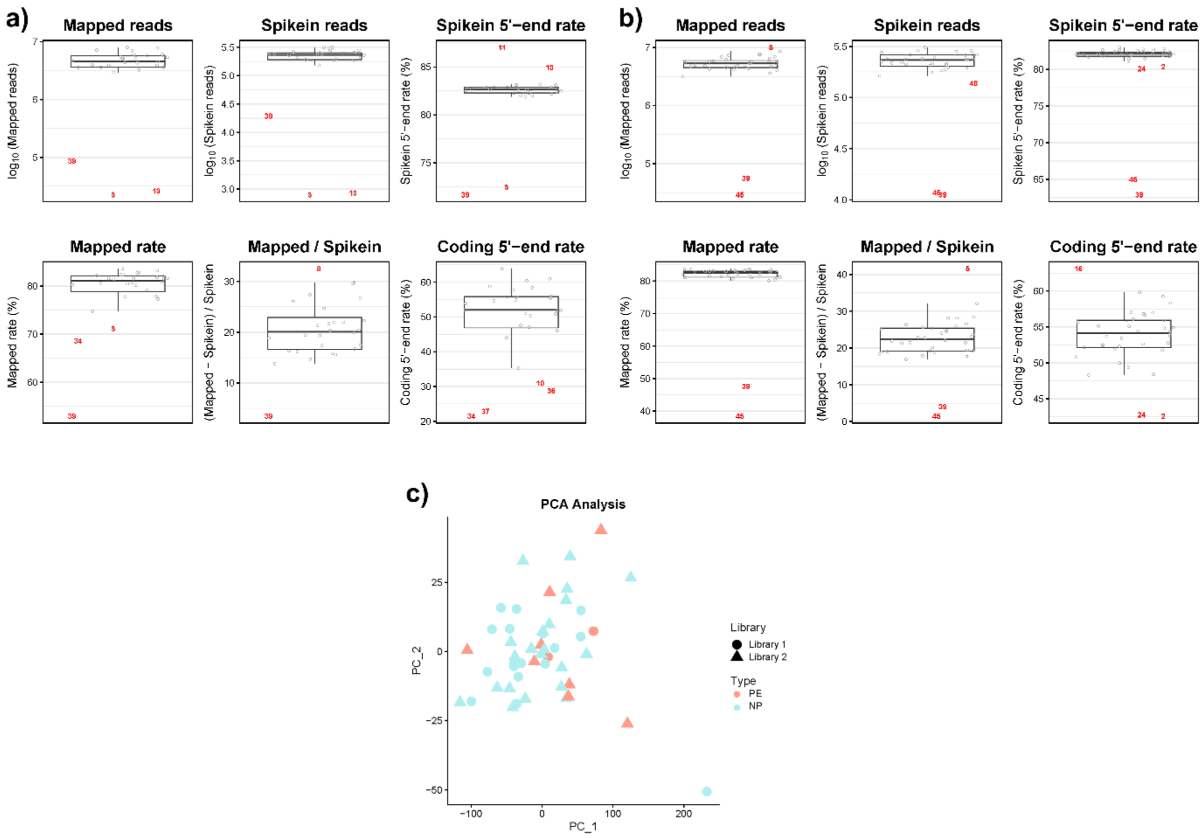

Supplementary Figure 1. Quality control and outlier detection in two STRT libraries. Outliers were identified based on the quality control metrics in both libraries. (a) In the first library, outliers were detected in samples 3, 8, 10, 11, 13, 15 (non-template control, NTC), 34, 36, and 39. (b) In the second library, outliers were observed in samples 2, 5, 11 (NTC), 16, 24, 39, 45, and 48. These outlier samples were excluded from further analysis. (c) PCA showing no bias between the STRT-seq libraries. PE indicates preeclampsia and NP normotensive pregnancy.

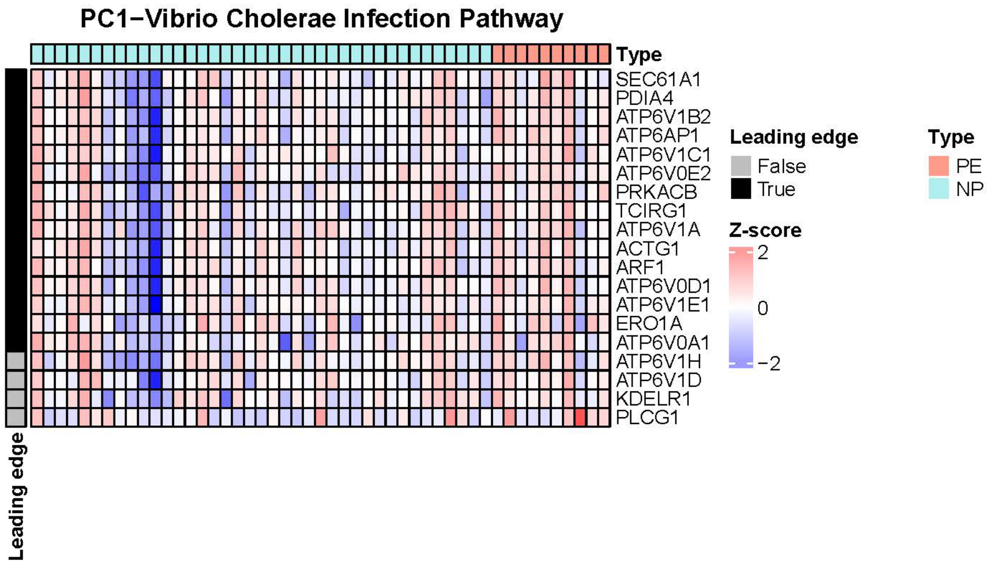

Supplementary Figure 2. Expression profiles of the genes in Vibrio cholerae infection pathway. Columns represent samples (ordered by rotated values in PC1), and rows represent pathway genes (ordered by loading values in PC1), with the left indicating leading genes. Red color indicates upregulation, and blue indicates downregulation, normalized across preeclampsia (PE; red) and normotensive pregnancies (NP; blue).

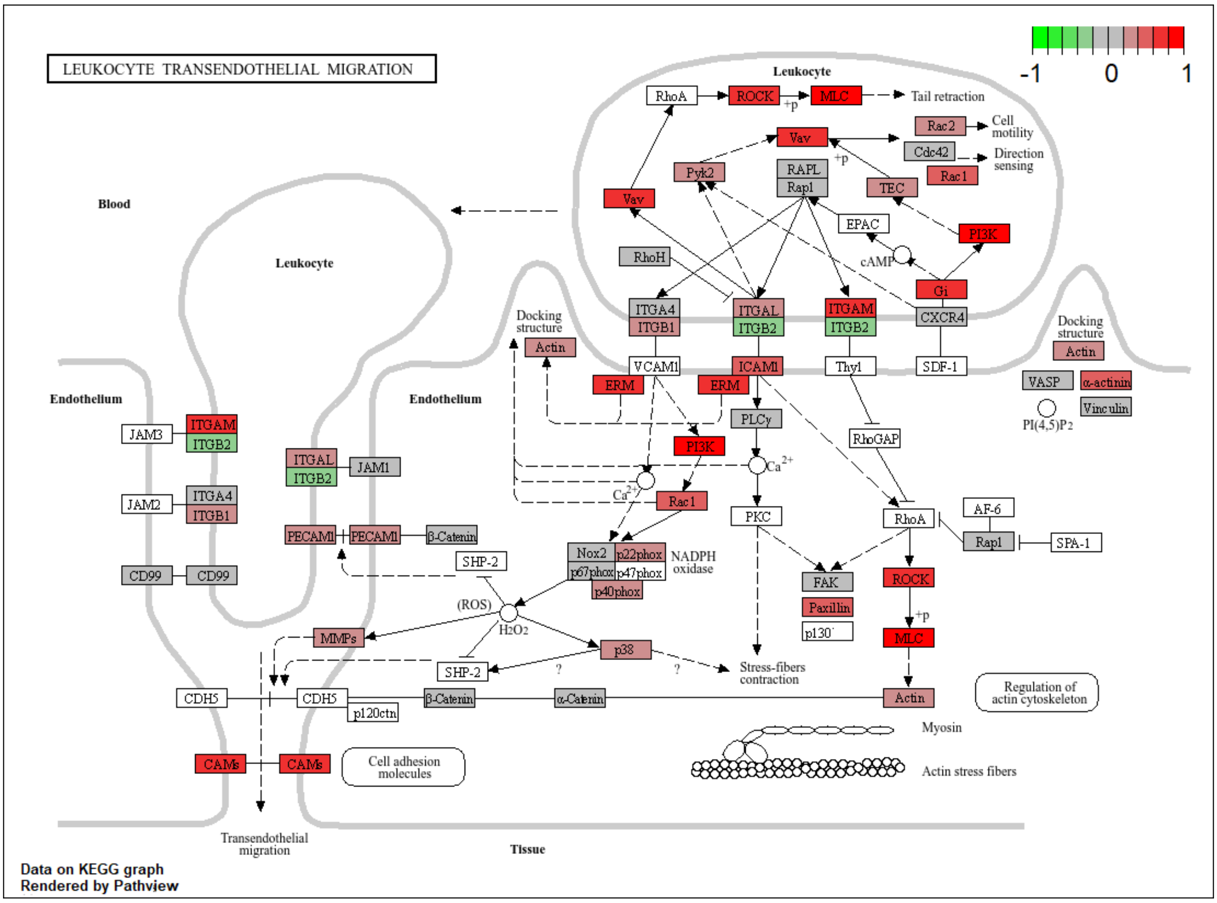

Supplementary Figure 3. KEGG Pathview graph of leukocyte transendothelial migration pathway. The log2FC of gene expressions in preeclampsia (PE) compared to normotensive pregnancy are shown. Red represents upregulation in PE, green represents downregulation, gray shows genes with no significant change, and white shows genes excluded during data cleaning.

Supplementary Figure 3 shows dysregulation of leukocyte transendothelial migration in PE, characterized by upregulation of ITGAM, PECAM1, ITGAL, ITGB1, ROCK, MLC, PI3K (red), indicating enhanced cell adhesion, integrin activation, and cytoskeletal remodeling, which may contribute to increased immune cell trafficking and inflammation. Conversely, downregulation of ITGB2 (green) suggests altered integrin signaling, potentially affecting immune cell movements. Pathway map reproduced from KEGG, Kanehisa Laboratories[1,2].

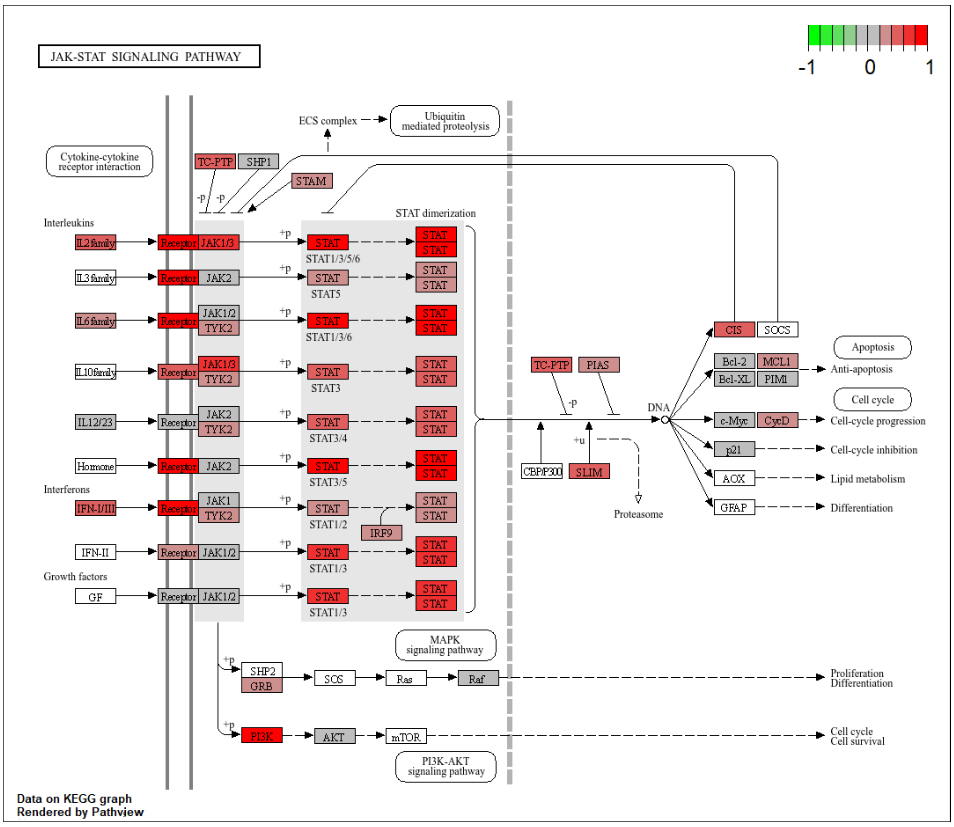

Supplementary Figure 4. KEGG Pathview graph of JAK-STAT signaling pathway. The log2FC of gene expressions in preeclampsia (PE) compared to normotensive pregnancy are shown. Red represents upregulation in PE, green represents downregulation, gray shows genes with no significant change, and white shows genes excluded during data cleaning.

Supplementary Figure 4 shows upregulated JAK-STAT signaling in PE, with upregulated genes (red) indicating increased activation of JAK kinases (JAK1, JAK2, JAK3, TYK2) and STAT transcription factors (STAT1, STAT3, STAT5, STAT6). This suggests increased cytokine signaling, inflammation and immune cell activation in PE. Additionally, PI3K-AKT and MAPK pathway activation suggests increased cell proliferation and survival in PE. Pathway map reproduced from KEGG, Kanehisa Laboratories[1,2].

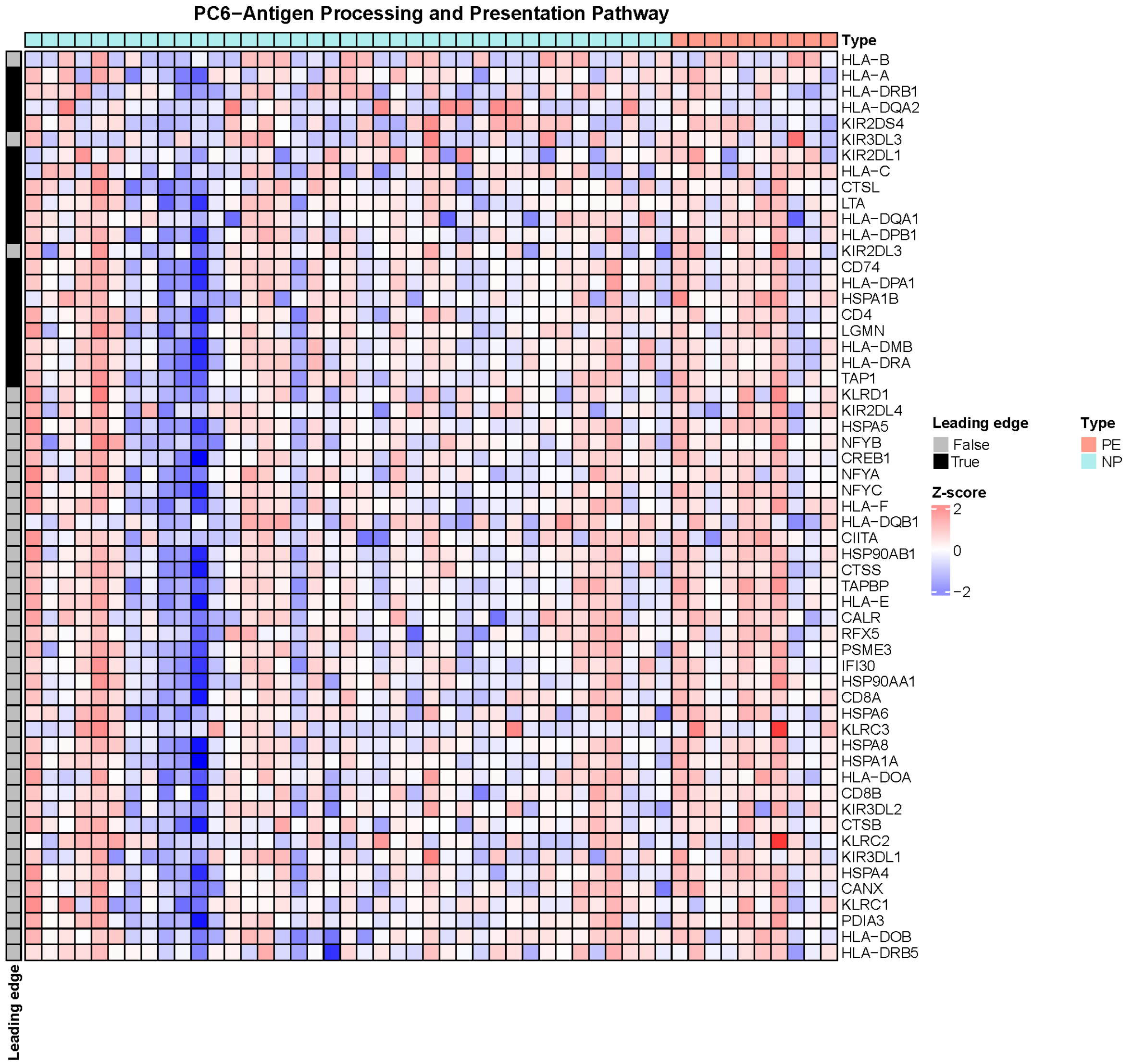

Supplementary Figure 5. Expression profiles of the genes in antigen processing and presentation pathway. Columns represent samples (ordered by rotated values in PC6), and rows represent pathway genes (ordered by loading values in PC6), with the left indicating leading genes. Red color indicates upregulation, and blue indicates downregulation, normalized across preeclampsia (PE; red) and normotensive pregnancies (NP; blue).

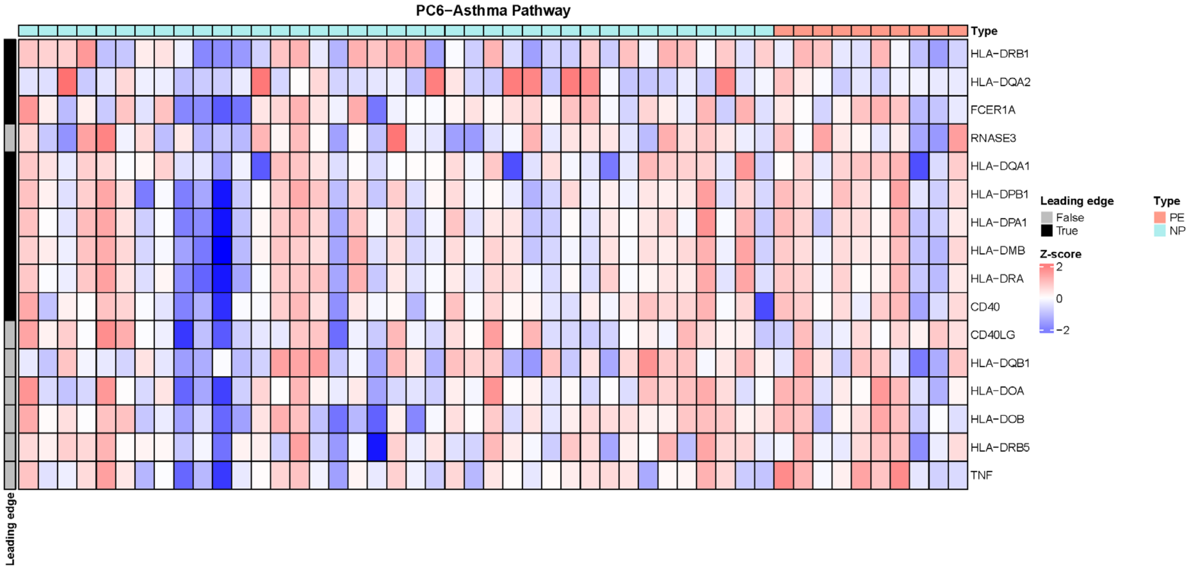

Supplementary Figure 6. Expression profiles of genes in asthma pathway. Columns represent samples (ordered by rotated values in PC6), and rows represent pathway genes (ordered by loading values in PC6), with the left indicating leading genes. Red color indicates upregulation, and blue indicates downregulation, normalized across preeclampsia (PE; red) and normotensive pregnancies (NP; blue).

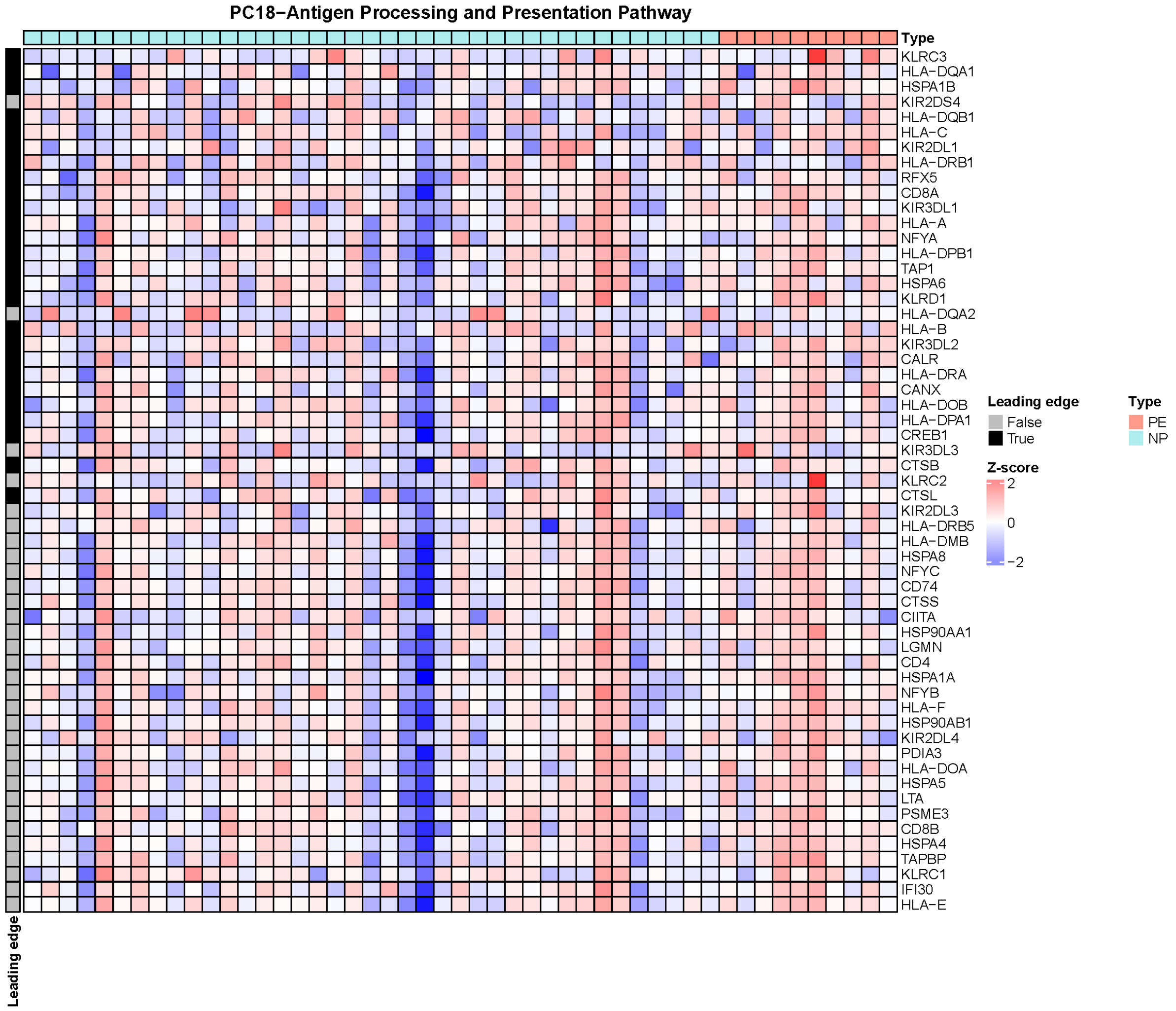

Supplementary Figure 7. Gene expression profile of genes in antigen processing and presentation pathway. Columns represent samples (ordered by rotated values in PC18), and rows represent pathway genes (ordered by loading values in PC18), with the left indicating leading genes. Red color indicates upregulation, and blue indicates downregulation, normalized across preeclampsia (PE; red) and normotensive pregnancies (NP; blue).

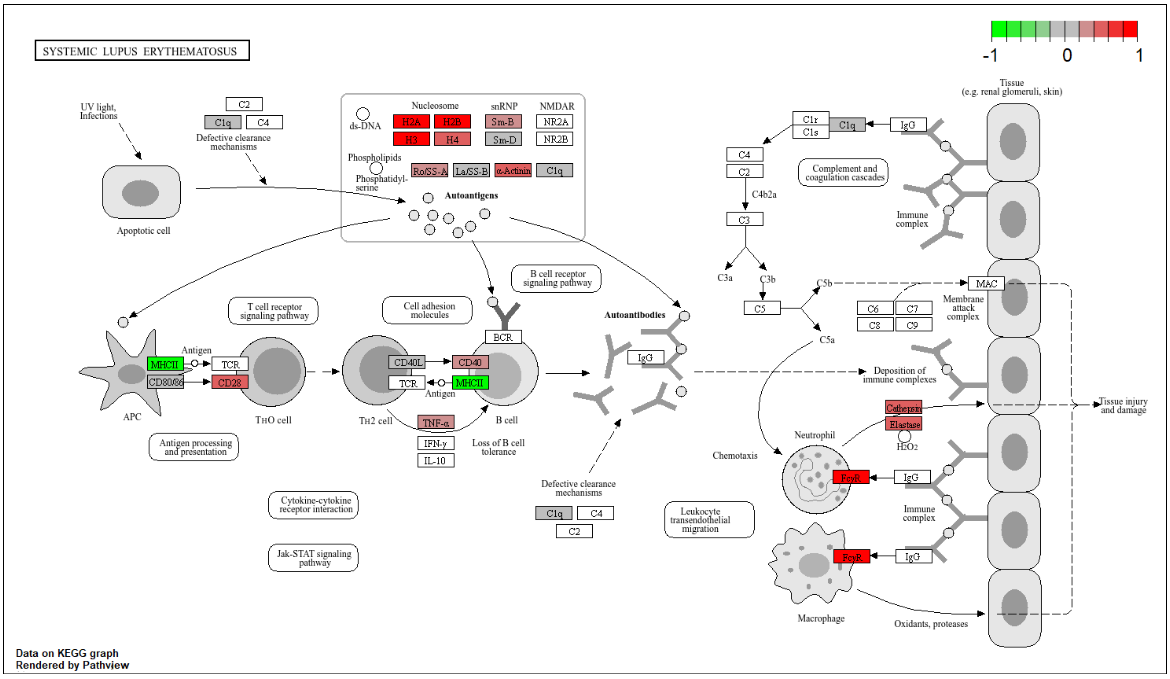

Supplementary Figure 8. KEGG Pathview graph of SLE pathway. The log2FC of gene expressions in preeclampsia (PE) compared to normotensive pregnancies (NP) are shown. Red represents upregulation in PE, green represents downregulation, gray shows genes with no significant change, and white shows genes excluded during data cleaning.

Supplementary Figure 8 shows immune dysregulation in PE with upregulated genes (red) indicating increased T cell activation (CD28, CD40), inflammatory cytokines (TNFα) and immune complex deposition (FcγR, cathepsin, elastase). Downregulation of MHC-II (green) suggests potential alterations in antigen presentation. These findings suggest increased immune activation and inflammatory processes in PE. Pathway map reproduced from KEGG, Kanehisa Laboratories[1,2].

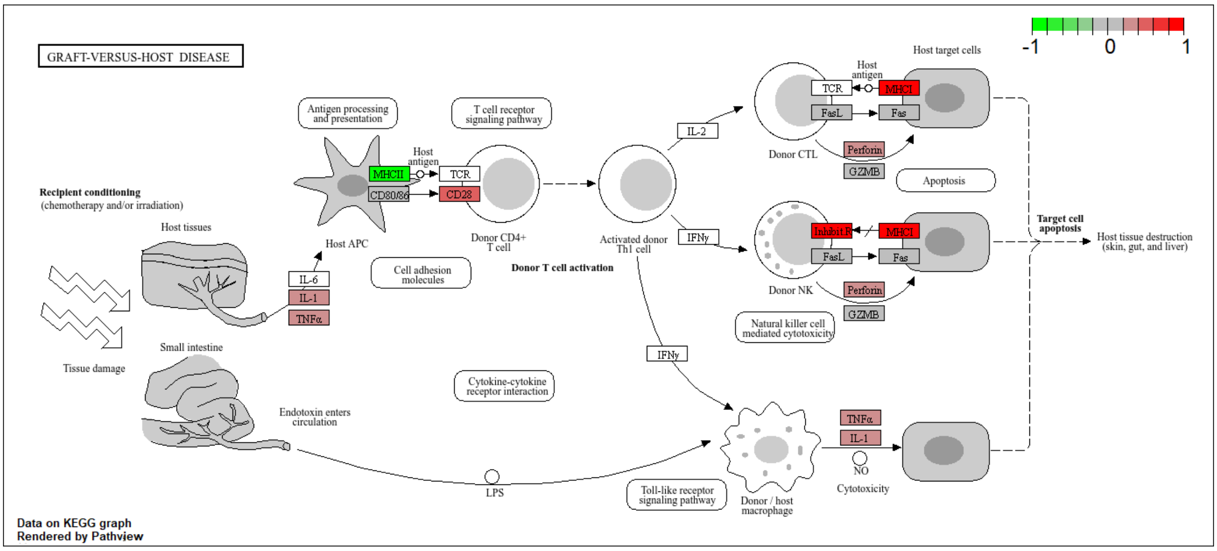

Supplementary Figure 9. KEGG Pathview graph of GVHD pathway. The log2FC of gene expressions in preeclampsia (PE) compared to normotensive pregnancies (NP) are shown. Red represents upregulation in PE, green represents downregulation, gray shows genes with no significant change, and white shows genes excluded during data cleaning.

Supplementary Figure 9 shows immune dysregulation in PE. Upregulated genes (red) indicate increased T cell activation, proinflammatory cytokines (TNFα, IL-1) suggesting excessive immune activation in PE. Additionally, the altered antigen presentation (MHC-II, CD28), suggests that dysregulation of immune signaling in PE. Pathway map reproduced from KEGG, Kanehisa Laboratories[1,2].

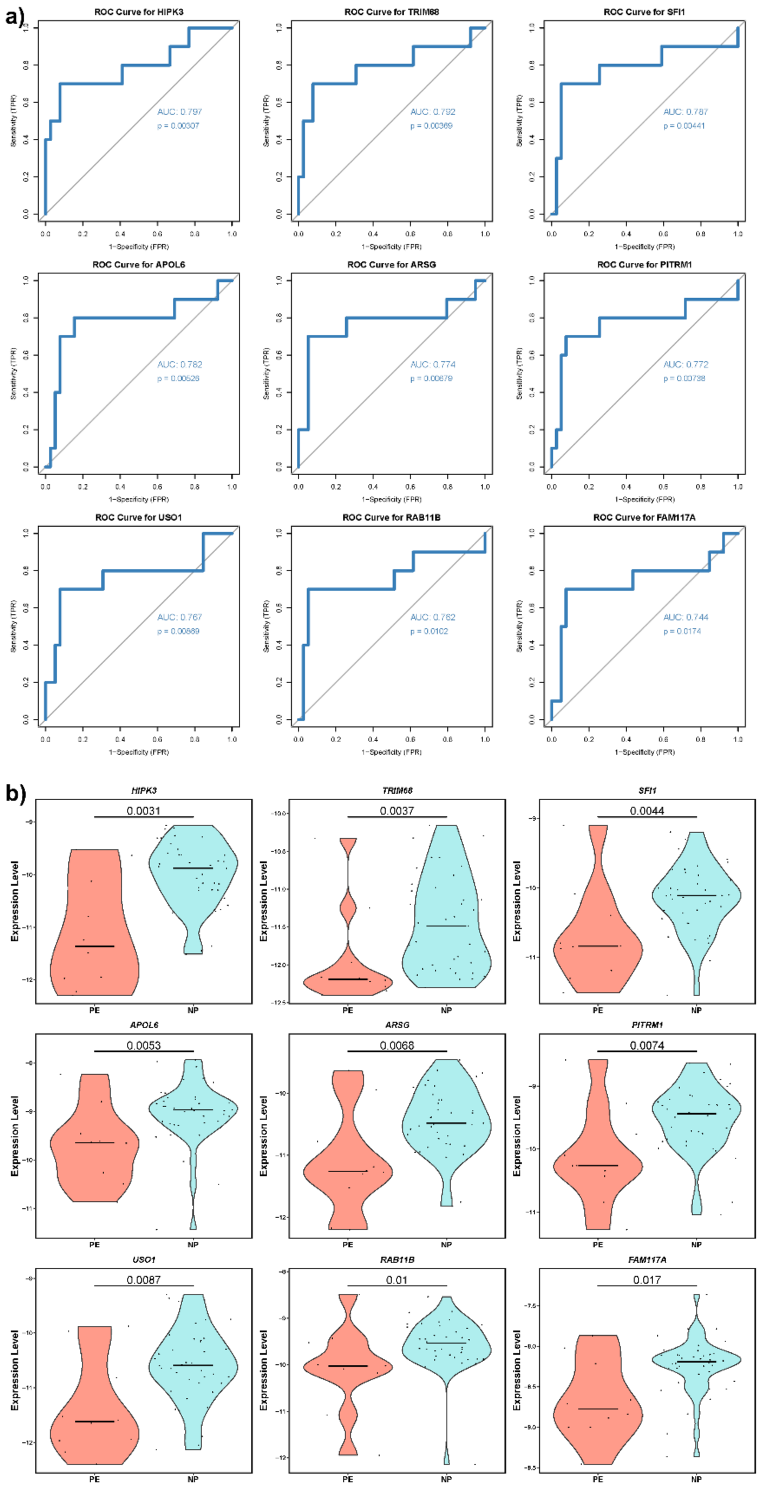

Supplementary Figure 10. Diagnostic performance of selected genes for preeclampsia prediction. (a) Receiver operating characteristic (ROC) curves showing the ability of biomarkers to distinguish preeclampsia (PE) from normotensive pregnancies (NP), with the area under curve (AUC) values and corresponding p-values (testing whether AUC>0.5). The false positive rate (FPR) is shown on the x-axis and true positive rate (TPR) on the y-axis. (b) Violin plots showing gene expression differences between PE and NP (two-sided Wilcoxon test p-values shown; horizontal lines indicate medians).

References

1. Kanehisa, M., Furumichi, M., Sato, Y., Matsuura, Y. & Ishiguro-Watanabe, M. KEGG: biological systems database as a model of the real world. *Nucleic Acids Research* **53**, D672–D677 (2025).
2. Kanehisa, M. & Goto, S. KEGG: Kyoto Encyclopedia of Genes and Genomes. *Nucleic Acids Res.* **28**, 27–30 (2000).
